# Uric Acid and Clinical Outcomes Among Intracerebral Hemorrhage Patients: Results From the China Stroke Center Alliance

**DOI:** 10.3389/fneur.2020.609938

**Published:** 2020-12-03

**Authors:** Xinmin Liu, Zhentang Cao, Hongqiu Gu, Kaixuan Yang, Ruijun Ji, Zixiao Li, Xingquan Zhao, Yongjun Wang

**Affiliations:** ^1^Department of Neurology, Beijing Tiantan Hospital, Capital Medical University, Beijing, China; ^2^China National Clinical Research Center for Neurological Diseases, Beijing, China; ^3^Center of Stroke, Beijing Institute for Brain Disorders, Beijing, China; ^4^Beijing Key Laboratory of Translational Medicine for Cerebrovascular Disease, Beijing, China

**Keywords:** uric acid, intracerebral hemorrhage, severity, mortality, complication

## Abstract

**Background and Purpose:** The effect of uric acid (UA) levels on severity and prognosis of spontaneous intracerebral hemorrhage (ICH) remains controversial. We aimed to explore the association of admission UA levels with stroke severity and outcomes in ICH patients.

**Materials and Methods:** The patients enrolled in this study were from the China Stroke Center Alliance study (CSCA). Patients were divided into four groups (Q1–Q4) according to the quartiles of UA levels at admission. The primary outcome was in-hospital mortality. The secondary outcomes included stroke severity, in-hospital complications, and discharge disposition. Multivariate logistic regression was adopted to explore the association of UA levels with outcomes after ICH.

**Results:** Patients (84,304) with acute ICH were included in the final analysis; the median (interquartile range) of UA was 277 (210, 354) μmol/L. The four groups were defined as follows: Q1 ≤ 210 μmol/L, 210 μmol/L < Q2 ≤ 277 μmol/L, 277 μmol/L < Q3 ≤ 354 μmol/L, Q4 > 354 μmol/L. There was no significant evidence indicating that UA levels were correlated with the discharge disposition and in-hospital mortality after ICH. However, compared to Q1, the patients with higher UA levels had decreased odds of severe stroke (NIHSS ≥ 16) at admission (OR 0.89, 95% CI 0.86–0.92). An L-shaped association was found between UA and severe stroke. Among in-hospital complications, decrease in pneumonia, poor swallow function, gastrointestinal bleeding, and deep vein thrombosis (DVT) were significantly associated with higher UA levels compared to Q1 (*P for trend* < 0.0001).

**Conclusions:** UA was a protective factor for stroke severity and in-hospital complications such as pneumonia, poor swallow function, gastrointestinal bleeding, and DVT. However, no significant evidence indicated that UA levels were predictive of the discharge disposition and in-hospital mortality after ICH.

## Introduction

Stroke remains the second leading cause of death and disability worldwide. Especially, the mortality and disability rate of intracerebral hemorrhage (ICH) is higher than other stroke types, of which the incidence rate is remarkable among the Asian population (1, 2). Due to the lack of specific treatments for ICH, it is imperative to improve approaches including efficacious neuroprotectants to lessen the burden of ICH worldwide.

Uric acid (UA) is the product of purine nucleotide catabolism. It has been proven that hyperuricemia was significantly correlated with several vascular risk factors such as hypertension, diabetes, and other metabolic diseases (3–6). In addition, increasing UA was associated with high morbidity, poor function outcome, and high mortality of atherosclerotic diseases, such as carotid artery disease, acute stroke, and cardiovascular disease (7–9). However, serum UA, with the capacity of free radical scavenging, has been considered as a free radical scavenger protecting nerves from oxidative damage (10, 11). Several studies have documented significant correlations between lower UA levels and poor outcome after acute stroke (12–14). The URICO-ICTUS study, a clinical trial of intravenous UA administered during alteplase treatment for acute ischemic stroke, found that UA therapy was excellently safe and may prevent early ischemic worsening after acute cerebral infarction among thrombolysed patients (15). Despite the existing and current studies, the neuroprotective effect of UA in stroke is still controversial.

Furthermore, the previous literatures were mainly limited to ischemic stroke rather than hemorrhagic stroke. Only a small amount of studies explored the effect of UA on ICH patients. It was a pity that they found no significant evidence indicating that UA levels were related to the severity and outcome after ICH (12, 16, 17). Their limitations, such as small sample size and single-center study, may need to be further investigated. Moreover, fewer studies have explored the relationship between UA and in-hospital mortality and complications in ICH patients.

Therefore, it is still unclear whether UA is an effective neuroprotectant to promote or protect against the poor outcome of ICH, or simply acts as a marker of increased risk. The aim of our study was to evaluate the association of admission UA with stroke severity, in-hospital mortality, discharge deposition, and in-hospital complications among ICH patients from the China Stroke Center Alliance.

## Materials and Methods

### Ethics

Participating hospitals received either healthcare quality assessment and research approval to collect data in the CSCA project without requiring individual patient informed consent under the common rule or a waiver of authorization and exemption from subsequent review by their Institutional Review Board. Patient confidentiality will be protected in the following ways: (1) data are stripped of all identifiers before their use in research and (2) the use of data for these purposes is closely overseen by the China National Clinical Research Center for Neurological Diseases analytic center.

### Study Design and Participants

We derived data from the China Stroke Center Alliance (CSCA), a national, hospital-based, multicenter, quality assessment, and improvement initiative performed in China (18). The inclusion criteria were as follows: age ≥ 18 years; primary diagnosis of acute stroke/TIA confirmed by brain CT or MRI, including ischemic stroke (IS), transient ischemic attack (TIA), intracerebral hemorrhage, or subarachnoid hemorrhage (SAH) within 7 days of symptom onset; admission either directly to wards or through the emergency department. As of July 2019, the trial enrolled 1,006,798 consecutive patients with acute stroke or TIA from 1,476 designed hospitals in China including 85,705 patients diagnosed with spontaneous ICH.

### Demographic and Clinical Information

Demographic information, body mass index (BMI), systolic blood pressure (SBP), diastolic blood pressure (DBP), smoking, alcohol use, medical history, and medication history were collected at admission. Fasting whole blood samples were taken into a vacutainer tube after admission. Afterward, serum UA was measured by automated hematology analyzer at each research center. The patients were divided into four groups (Q1–Q4) according to the quartiles of UA levels. Other laboratory tests, including low-density lipoprotein cholesterol (LDL-C), glycated hemoglobin (GHb), fasting blood glucose (FBG), homocysteine (Hcy), creatinine (Cr), blood urea nitrogen (BUN), and platelets (PLT) were also collected.

### Outcome

The primary outcome was in-hospital mortality. The secondary outcomes included stroke severity, discharge deposition, and in-hospital complications. Stroke severity was assessed by the National Institutes of Health Stroke Scale (NIHSS) score at admission. Severe stroke was defined as NIHSS ≥ 16. Discharge disposition was categorized into routine or non-routine. Discharging to home was considered to be routine disposition, while discharging to graded II or III hospital, community hospital, or rehabilitation facilities were considered as non-routine disposition. The in-hospital complications included pneumonia, poor swallow function, gastrointestinal bleeding, and deep vein thrombosis (DVT).

### Statistical Analysis

Baseline clinical features and outcomes were compared stratified by quartile levels of UA. Categorical variables were described by frequencies with percentages. We used the Kolmogorov Smirnov test to evaluate the normal distribution data of continuous variables. Continuous variables of skewed distribution were described by medians with interquartile ranges. If the distribution was normal, the continuous variables were presented as mean ± standard deviation. For the continuous variables, if the data were normally distributed, the comparisons were used in one-way ANOVA test; otherwise, the Mann–Whitney *U*-test was used instead. For categorical variables, the Chi-square test was used to perform the comparison.

Multivariable logistic regression analysis based on two models was used to determine the association of admission UA levels with stroke severity, in-hospital complications, discharge deposition, and in-hospital mortality. In the first model, we adjusted only the age and sex. In the second model, we included all the potential covariates with *p*-value < 0.05. In addition, we further performed a logistic regression model with restricted cubic splines for UA for the severe stroke. The five knots for spline were placed at the 5, 25, 50, 75, and 95th percentiles of UA levels.

A two-sided *p*-value < 0.05 was considered to be statistically significant. All analyses were performed with the SAS software version 9.4 (SAS Institute Inc., Cary, NC, USA).

## Results

### Baseline Characteristics

There were 85,705 spontaneous ICH patients enrolled in the CSCA. After excluding 141 patients missing in-hospital mortality data and 1,260 patients missing UA data, 84,304 patients were involved in the current analysis. Patients were divided into four groups (Q1–Q4) according to the quartiles of UA levels, defined as follows: Q1 ≤ 210 μmol/L, 210 μmol/L < Q2 ≤ 277 μmol/L, 277 μmol/L < Q3 ≤ 354 μmol/L, Q4 > 354 μmol/L.

The differences in the clinical characteristics of patients among different UA level groups are shown in Tables 1, 2. Q4 had the highest percentage of males (*P* < 0.0001), and the mean age in this group was significantly younger (*P* < 0.0001). Meanwhile, there was higher prevalence of alcohol use and current smoking among Q4 (*P* < 0.0001). The patients of Q4 were also more likely to have higher proportions of history of TIA, hypertension, diabetes mellitus, dyslipidemia, atrial fibrillation, heart failure, and peripheral vascular disorder (*P* < 0.0001). In physical examination and laboratory tests, the patients in the Q4 group had significantly higher blood pressure, BMI, LDL-C, GHb, FBG, Hcy, Cr, and BUN (all *P* < 0.01). In terms of in-hospital outcome, the mean length of stay was longer, and hospital expenditure was significantly higher in the Q1 group (*P* < 0.0001).

**Table 1:** Baseline characteristics among intracerebral hemorrhage (ICH) patients in China Stroke Center Alliance (CSCA).

**Variables**	**Total (*n* = 84,304)**	**Q1** **(*n* = 21,196)**	**Q2** **(*n* = 21,117)**	**Q3** **(*n* = 21,046)**	**Q4** **(*n* = 20,945)**	***P*-value**
**Demographics**						
Age, y, mean (SD)	62.9 ± 12.9	63.7 ± 12.7	63.7 ± 12.3	63.0 ± 12.7	61.2 ± 13.6	<0.0001
Male, n (%)	52,693 (62.5)	9,832 (46.4)	11,750 (55.6)	14,539 (69.1)	16,572 (79.1)	<0.0001
**Physical examination, mean (SD)**						
BMI, kg/m^2^	23.9 ± 4.4	23.7 ± 4.5	23.7 ± 3.8	23.9 ± 4.2	24.3 ± 5.1	<0.0001
SBP, mmHg	164.6 ± 28.2	162.6 ± 27.7	163.7 ± 27.4	164.7 ± 28.0	167.4 ± 29.5	<0.0001
DBP, mmHg	95.3 ± 16.9	93.2 ± 16.2	94.4 ± 16.0	95.6 ± 16.5	98.0 ± 18.2	<0.0001
**Medical history**, ***n*** **(%)**						
TIA	514 (0.6)	121 (0.6)	120 (0.6)	136 (0.6)	137 (0.7)	<0.0001
Ischemic stroke	10,978 (13.0)	2,695 (12.7)	2,942 (13.9)	2,842 (13.5)	2,499 (11.9)	<0.0001
SAH	458 (0.5)	136 (0.6)	115 (0.5)	101 (0.5)	106 (0.5)	0.1168
ICH	14,957 (17.7)	4,204 (19.8)	3,670 (17.4)	3,497 (16.6)	3,586 (17.1)	<0.0001
Hypertension	60,152 (71.4)	14,350 (67.7)	14,954 (70.8)	15,195 (72.2)	15,653 (74.7)	<0.0001
DM	8,021 (9.5)	1,985 (9.4)	2,016 (9.5)	1,972 (9.4)	2,048 (9.8)	<0.0001
Dyslipidemia	3,568 (4.2)	780 (3.7)	825 (3.9)	862 (4.1)	1,101 (5.3)	<0.0001
Atrial fibrillation	1,285 (1.5)	250 (1.2)	279 (1.3)	326 (1.5)	430 (2.1)	<0.0001
Heart failure	396 (0.5)	72 (0.3)	77 (0.4)	93 (0.4)	154 (0.7)	<0.0001
Myocardial infarction	754 (0.9)	182 (0.9)	185 (0.9)	189 (0.9)	198 (0.9)	<0.0001
PVD	816 (1.0)	194 (0.9)	178 (0.8)	214 (1.0)	230 (1.1)	<0.0001
**Behavioral history**, ***n*** **(%)**						
Current smoking	16,591 (19.7)	2,783 (13.1)	3,631 (17.2)	4,662 (22.2)	5,515 (26.3)	<0.0001
Drinking	20,561 (24.4)	3,776 (17.8)	4,342 (20.6)	5,578 (26.5)	6,865 (32.8)	<0.0001
**Medication history**, ***n*** **(%)**						
Antiplatelet	5,774 (6.8)	1,469 (6.9)	1,505 (7.1)	1,482 (7.0)	1,318 (6.3)	0.0015
Anticoagulation	1,593 (1.9)	474 (2.2)	344 (1.6)	336 (1.6)	439 (2.1)	<0.0001
Antihypertensive	39,776 (47.2)	9,507 (44.9)	9,882 (46.8)	10,038 (47.7)	10,349 (49.4)	<0.0001
Diabetic medication	5,869 (7.0)	1,464 (6.9)	1,471 (7.0)	1,434 (6.8)	1,500 (7.2)	<0.0001
Cholesterol-reduce	4,860 (5.8)	1,201 (5.7)	1,188 (5.6)	1,200 (5.7)	1,271 (6.1)	0.0055
**Laboratory test, mean (SD)**						
LDL-C, mmol/L	2.8 ± 1.6	2.8 ± 1.9	2.7 ± 1.3	2.7 ± 1.3	3.0 ± 1.7	<0.0001
GHb, mmol/L	5.9 ± 1.7	5.9 ± 1.8	5.9 ± 1.6	5.9 ± 1.6	6.0 ± 1.9	<0.0001
FBG, mmol/L	6.5 ± 2.8	6.5 ± 2.9	6.5 ± 2.7	6.5 ± 2.6	6.7 ± 3.2	<0.0001
Hcy, mmol/L	14.0 ± 8.1	13.1 ± 8.3	13.7 ± 7.7	14.2 ± 7.9	15.0 ± 8.3	<0.0001
Cr, mmol/L	135.0 ± 1,288.9	98.6 ± 881.4	91.3 ± 551.6	114.0 ± 897.6	236.9 ± 2184.3	<0.0001
BUN, mmol/L	5.7 ± 2.8	5.1 ± 2.6	5.4 ± 2.2	5.7 ± 2.4	6.7 ± 3.5	<0.0001
PLT, 10^9^/L	203.2 ± 70.3	203.3 ± 72.3	204.4 ± 68.3	202.6 ± 68.7	202.4 ± 71.9	0.0051

*ICH, intracerebral hemorrhage; BMI, body mass index; SBP, systolic blood pressure; DBP, diastolic blood pressure TIA, transient ischemic attack; SAH, subarachnoid hemorrhage; DM, diabetes mellitus; PVD, peripheral vascular disorder; LDL-C, low-density lipoprotein cholesterol; GHb, glycated hemoglobin; FBG, fasting blood glucose; Hcy, homocysteine; Cr, creatinine; BUN, blood urea nitrogen; PLT, platelets*.

**Table 2:** Clinical characteristics and severity and in-hospital outcome in ICH patients.

**Variables**	**Q1**	**Q2**	**Q3**	**Q4**	***P*-value**
**NHISS, median (IQR)**	7.0 (2.0–14.0)	5.0 (2.0–12.0)	5.0 (2.0–11.0)	5.0 (2.0–12.0)	<0.0001
**GCS, median (IQR)**	12.0 (7.0–15.0)	14.0 (8.0–15.0)	14.0 (9.0–15.0)	14.0 (8.0–15.0)	<0.0001
**Severe stroke**, ***n*** **(%)**	2,314 (20.4)	1,995 (15.8)	1,826 (14.4)	2,017 (16.7)	<0.0001
**In-hospital complications**, ***n*** **(%)**					
Rebleeding	1,688 (8.0)	1,708 (8.1)	1,650 (7.9)	1,927 (9.2)	<0.0001
Pneumonia	6,399 (30.2)	5,003 (23.7)	4,886 (23.3)	5,305 (25.4)	<0.0001
Poor swallow function	3,737 (26.5)	3,204 (21.1)	3,178 (20.7)	3,364 (22.3)	<0.0001
Pulmonary embolism	87 (0.4)	51 (0.2)	44 (0.2)	44 (0.2)	<0.0001
Seizure	280 (1.3)	286 (1.4)	248 (1.2)	371 (1.8)	<0.0001
Urinary tract infection	602 (2.8)	505 (2.4)	493 (2.3)	490 (2.3)	0.0016
Hydrocephalus	549 (2.6)	448 (2.1)	397 (1.9)	446 (2.1)	<0.0001
DVT	352 (1.7)	302 (1.4)	227 (1.1)	239 (1.1)	<0.0001
Decubitus	157 (0.7)	120 (0.6)	116 (0.6)	128 (0.6)	0.0560
Gastrointestinal bleeding	634 (3.0)	566 (2.7)	566 (2.7)	656 (3.1)	0.0092
**Hematoma evacuation**, ***n*** **(%)**	3,081 (15.2)	1,977 (9.8)	1,855 (9.3)	1,874 (9.4)	<0.0001
**In-hospital mortality**, ***n*** **(%)**	452 (2.1)	395 (1.9)	424 (2.0)	675 (3.2)	<0.0001
**Length of hospital stay, mean (SD)**	17.8 ± 12.5	16.6 ± 11.0	16.2 ± 11.1	15.9 ± 11.9	<0.0001
**Hospital expenditure, RMB, mean (SD)**	20,955.3 ± 19,031.7	17,552.6 ± 16,112.0	17,233.7 ± 15,847.3	17,848.5 ± 16,714.7	<0.0001
**Non-routine disposition**, ***n*** **(%)**	1,999 (10.0)	1,630 (8.0)	1,688 (8.4)	1,872 (9.5)	<0.0001

*NIHSS, National Institute of Health stroke scale; GCS, Glasgow coma scale; DVT, deep vein thrombosis*.

### Correlation of Serum UA With In-hospital Mortality and Discharge Disposition

As shown in Table 3, after adjusting only for age and sex, there was a negative correlation between the UA levels and the incidence of non-routine disposition. The ORs compared to Q1 were 0.80 (95% CI 0.76–0.85), 0.81 (95% CI 0.77–0.85), and 0.93 (95% CI 0.88–0.98) from Q2 to Q4. Meanwhile, the regression showed that the ORs for the association of Q2 and Q4 with occurrence rate of in-hospital mortality were 0.87 (95% CI 0.76–1.00) and 1.57 (95% CI 1.39–1.78), respectively, but no significant difference was observed for Q3 (*P* = 0.3791). However, after introducing age, sex, SBP, DBP, BMI, current smoking, drinking, medication history, medical history, laboratory test, and in-hospital complications with a *P*-value < 0.05 in the univariate analysis in Tables 1, 2, and NIHSS, GCS, hematoma evacuation, length of hospital stay, and hospital expenditure into the regression model, there was no statistically significant difference indicating that UA levels were correlated with the non-routine disposition and in-hospital mortality after ICH.

**Table 3:** Logistic regression of the UA levels on discharge disposition and in-hospital mortality.

**Variable**	Model 1*		Model 2**	
	**OR(95% CI)**	***P*-value**	**OR(95% CI)**	***P*-value**
**Non-routine disposition**				
Q1	1	Reference	1	Reference
Q2	0.80 (0.76, 0.85)	<0.0001	0.95 (0.82, 1.09)	0.4371
Q3	0.81 (0.77, 0.85)	<0.0001	0.90 (0.78, 1.03)	0.1375
Q4	0.93 (0.88, 0.98)	0.0043	0.99 (0.86, 1.14)	0.8827
**In-hospital mortality**				
Q1	1	Reference	1	Reference
Q2	0.87 (0.76, 1.00)	0.0465	0.83 (0.49, 1.41)	0.4974
Q3	0.94 (0.82, 1.08)	0.3791	1.05 (0.63, 1.75)	0.8377
Q4	1.57 (1.39, 1.78)	<0.0001	1.56 (0.96, 2.52)	0.0696

Model 1* adjusted for age and sex. Model 2** adjusted for age, sex, SBP, DBP, BMI, current smoking, drinking, medication history, medical history, laboratory test, and in-hospital complications with a P-value < 0.05 in the univariate analysis in Tables 1, 2, and NIHSS, GCS, hematoma evacuation, length of hospital stay, and hospital expenditure.

### Correlation of Serum UA With Stroke Severity

As shown in Table 1, there was higher proportion of severe stroke (NIHSS ≥ 16) in the Q1 group. In the multivariate logistic regression analysis, after adjustment for age and sex, compared to the lowest quartile, UA was negatively associated with severe stroke with adjusted ORs of 0.94 (95% CI 0.92–0.96) (*P for trend* < 0.0001) (Table 4). When adjusted for age, sex, SBP, DBP, BMI, current smoking, drinking, medication history, medical history, and laboratory test with a *P*-value < 0.05 in the univariate analysis in Table 1, we found the same phenomenon that higher UA levels were significantly negatively correlated with severe stroke (OR 0.89, 95% CI 0.86–0.92, *P for trend* < 0.0001) (Table 4).

**Table 4:** Logistic regression of the uric acid levels on stroke severity.

**Variable**	Model 1*		Model 2**	
	**OR(95% CI)**	***P*-value**	**OR(95% CI)**	***P*-value**
**Severe stroke**				
Q1	1	Reference	1	Reference
Q2	0.75 (0.70, 0.80)	<0.0001	0.76 (0.70, 0.83)	<0.0001
Q3	0.69 (0.64, 0.74)	<0.0001	0.68 (0.62, 0.74)	<0.0001
Q4	0.85 (0.79, 0.91)	<0.0001	0.71 (0.65, 0.78)	<0.0001
*P* for trend	<0.0001		<0.0001	
Continuous scale	0.94 (0.92, 0.96)		0.89 (0.86, 0.92)	

Model 1^*^ adjusted for age and sex, Model 2^**^ adjusted for age, sex, SBP, DBP, BMI, current smoking, drinking, medication history, medical history, and laboratory test with a P-value < 0.05 in the univariate analysis in Table 1.

**Figure 1:**
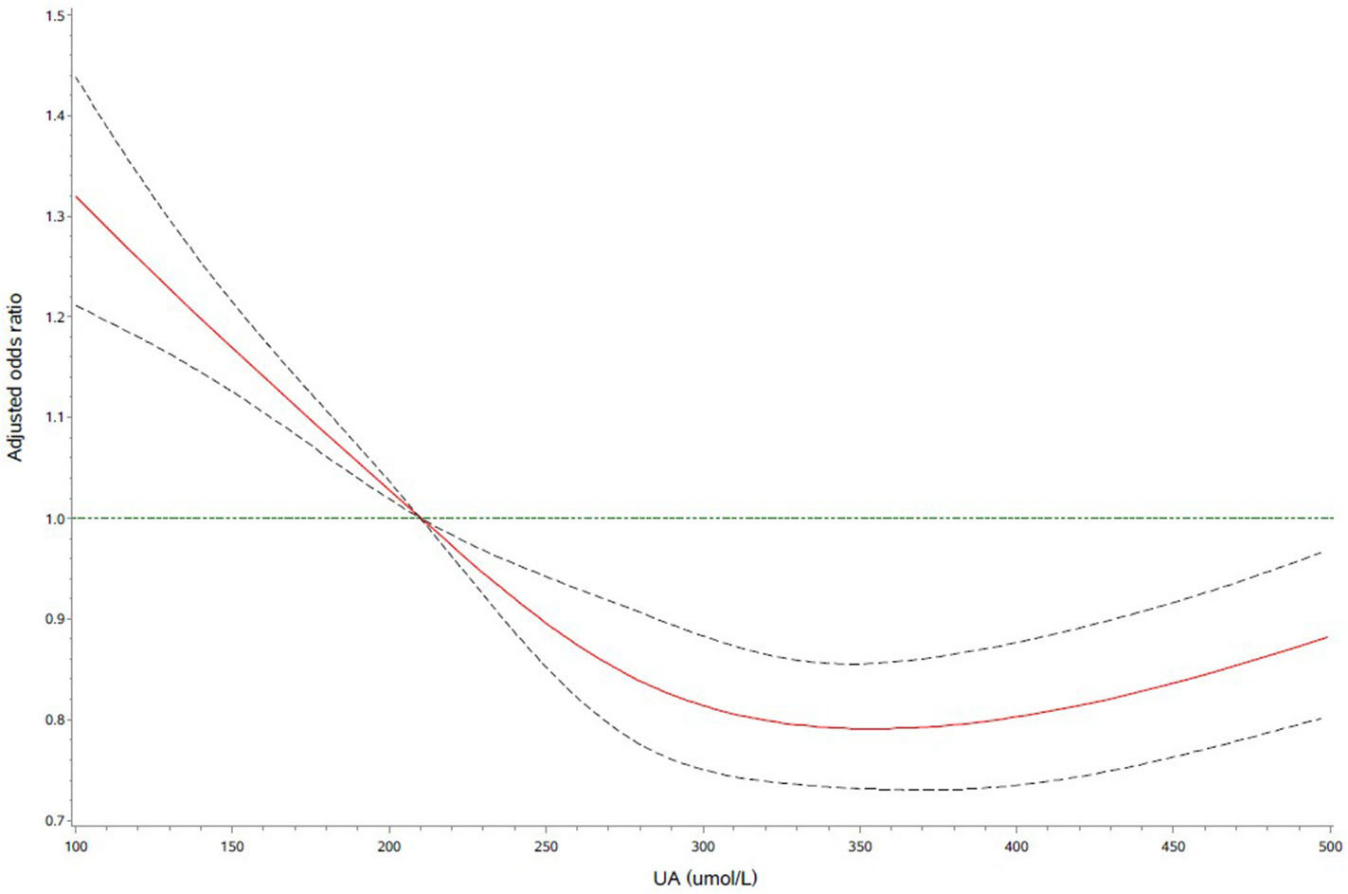
Adjusted odds ratio of severe stroke according to uric acid (UA). Red lines indicate adjusted hazard/odds ratio, and black dotted line indicate the 95% confidence interval bands. Reference is UA of 210 μmol/L. Data were fitted with a logistic regression model of restricted cubic spline with five knots (5, 25, 50, 75, and 95th percentiles) for UA, with adjustment for age, sex, systolic blood pressure (SBP), diastolic blood pressure (DBP), body mass index (BMI), current smoking, drinking, medication history, medical history, and laboratory test with a *P*-value < 0.05 in the univariate analysis in Table 1. The lowest 5% and highest 5% of participants were not shown for the small sample sizes.

Further logistic regression analyses with restricted cubic spline indicated that higher UA levels were significantly associated with a decrease risk of severe stroke (Figure 1). What is more, we observed an L-shaped association between the UA levels and the risk of severe stroke. In the restricted cubic spline model, the risk of severe stroke was relatively flat when UA > 210 μmol/L.

### Correlation of Serum UA With In-hospital Complications

In multivariate logistic regression analysis, after adjustment for age, sex, SBP, DBP, BMI, current smoking, drinking, medication history, medical history, and laboratory test with a *P*-value < 0.05 in the univariate analysis in Table 1 and NIHSS at admission, the correlation of UA levels with various in-hospital complications was demonstrated. As shown in the Table 5, incidence of pneumonia, gastrointestinal bleeding, poor swallow function, and were significantly negatively associated with the higher UA levels. Compared to Q1, the ORs for incidence of pneumonia, poor swallow function, gastrointestinal bleeding, and DVT were 0.91 (95% CI 0.89–0.93), 0.93 (95% CI 0.91–0.95), 0.90 (95% CI 0.86–0.95), and 0.85 (95% CI 0.80–0.92), respectively (all *P for trend* < 0.0001).

**Table 5:** Multivariable regression models evaluating the correlation of UA with in-hospital complications.

**In-hospital complications**	**OR(95%CI)**	***P*-value**	***P* for trend**	**Continuous scale**
**Pneumonia**			<0.0001	0.91 (0.89, 0.93)
Q1	Reference			
Q2	0.74 (0.69, 0.78)	<0.0001		
Q3	0.70 (0.66, 0.74)	<0.0001		
Q4	0.76 (0.71, 0.81)	<0.0001		
**Poor swallow function**			<0.0001	0.93 (0.91, 0.95)
Q1	Reference			
Q2	0.77 (0.71, 0.82)	<0.0001		
Q3	0.75 (0.70, 0.81)	<0.0001		
Q4	0.80 (0.74, 0.86)	<0.0001		
**Gastrointestinal bleeding**			0.0001	0.90 (0.86, 0.95)
Q1	Reference			
Q2	0.80 (0.68, 0.93)	0.0031		
Q3	0.70 (0.60, 0.82)	<0.0001		
Q4	0.76 (0.65, 0.89)	0.0005		
**DVT**			<0.0001	0.85 (0.80, 0.92)
Q1	Reference			
Q2	0.88 (0.72, 1.07)	0.1945		
Q3	0.66 (0.53, 0.82)	0.0001		
Q4	0.66 (0.53, 0.82)	0.0002		

*DVT, deep vein thrombosis. Adjusted for age, sex, BMI, current smoking, drinking, medication history, medical history, and laboratory test with a p-value < 0.05 in the univariate analysis in Table 1 and NIHSS at admission*.

## Discussion

In the present study, the higher UA levels (UA > 210 μmol/L) were significantly and independently a protective factor for stroke severity and in-hospital complications such as pneumonia, poor swallow function, gastrointestinal bleeding, and DVT among ICH patients. Furthermore, we found an L-shaped association between the UA levels and the risk of severe stroke. However, there was no statistically significant correlation between UA levels and in-hospital mortality as well as discharge disposition in hemorrhagic stroke.

UA is the product of purine nucleotide catabolism, higher in men, postmenopausal women, renal diseases, the metabolic syndrome, alcohol drinkers, and those in a high purine diet (19). Consistently, UA was higher in males and alcoholics in our study. The patients in the highest UA level group was younger. The mean age of the total study population was over 60 years old. With the increase in age, UA levels in the elderly will decrease due to the low metabolic rate. Contradicted to our study, previous studies demonstrated that plasma UA concentration was significantly lower in smokers (20). That might be because we did not adjust the confounding factors such as history of alcohol consumption, diabetes mellitus, and so on. The patients of highest UA level were also more likely to have higher proportions of history of TIA, hypertension, diabetes mellitus, dyslipidemia, atrial fibrillation, heart failure, and peripheral vascular disorder. In physical examination and laboratory tests, the blood pressure, BMI, LDL-C, GHb, FBG, Hcy, Cr, and BUN were also significantly higher in the highest UA level group. This was because hyperuricemia was a risk factor for metabolic disease such as hypertension, dyslipidemia, and diabetes and further aggravated atherosclerosis. Hyperuricemia, renal disease, and cardiovascular disease are comorbid diseases (3–6).

The previous researches on the correlation between UA and stroke mainly focused on ischemic stroke or both ischemic stroke and hemorrhagic stroke. Several studies found that lower uric acid levels predicted good outcomes in acute stroke. Chamorro et al. demonstrated that there was a 12% increase in the odds of good clinical outcome for each milligram per deciliter increase in serum uric acid among acute ischemic stroke patients (21). Wu et al. showed that lower UA level (<221 μmol/L) was found to be at a significantly higher risk for the poor functional outcome (mRS > 2) at discharge in acute stroke with normoglycemia other than diabetes or prediabetes (14). Song et al. concluded that higher serum UA was independently correlated with lower hemorrhagic transformation following acute ischemic stroke (22). According to the protective effect of uric acid in acute ischemic stroke demonstrated in these observational studies, scientists further carried out interventional studies. The URICO-ICTUS clinical trials found that UA therapy was excellently safe and may prevent early ischemic worsening after acute ischemic stroke in thrombolysed patients (15, 16). However, limited researches focused only on acute ICH patients. Our findings on correlation of UA with the clinical outcomes after ICH provided evidence for whether UA could be used as a predictor or treatment of ICH and would inspire the interventional researches in future.

Rare literature explored the correlation between UA levels and stroke severity or in-hospital complications among ICH patients. We found that admission UA level was the independent indicator of initial stroke severity. The lower UA levels were significantly correlated with severe hemorrhagic stroke. However, in a prospective cohort of 43 patients with acute ischemic stroke, Kurzepa et al. found that average UA levels in hospital caused lower improvement of neurological state evaluated on day 10 after the stroke (23). Unlike the research, subjects in our study were only ICH patients, and the variables of the study did not involve dynamic changes in values but the admission UA levels and initial stroke severity. Moreover, heterogeneous clinical outcome assessments could partly explain the difference. UA is a natural antioxidant and an anti-inflammation factor, which could also stabilize the endothelium of cells (10, 24–26). In the acute stage of cerebral hemorrhage, UA might also play a role of neuroprotection and anti-inflammation (27). Brouns et al. demonstrated that a decrease in UA concentrations from admission to day 7 was larger in patients with initial severe ischemic stroke (NIHSS > 7) (28). The decrease in UA concentrations in acute stroke may reflect oxidative stress, which was influenced by stroke severity. It could be the theoretical mechanism of the results we found that lower UA levels were independent indicators of severe stroke. Thus, after onset of acute stroke, the level of UA was unstable but dynamically decreasing. Observational studies could only assess UA at a single time point. Interventional studies of administrating exogenous uric acid were warranted to investigate the impact of stable UA levels *in vivo* on clinical outcomes after ICH.

Furthermore, we found a decrease in complications such as pneumonia, poor swallow function, gastrointestinal bleeding, and DVT among ICH patients with higher UA levels. Dysphagia was a risk factor of aspiration pneumonia (29, 30). UA could reduce the incidence of pneumonia, malnutrition, and poor resistance by improving swallowing function. Unlike our study, in a clinical cohort study, hyperuricemia was proven to be an independent risk factor of colonic diverticular bleeding (31, 32), and the patients with gout were found to have a 1.38-fold (95% CI 1.18–1.62, *p* < 0.001) higher risk of developing DVT (33). Arteriosclerosis was the main cause of colonic diverticular bleeding, which was a chronic pathological state associated with UA (32). However, oxidative stress, defined as the main cause of gastrointestinal bleeding in the acute stage, could be alleviated by UA. The lower UA levels were associated with severe stroke, which tended to accompany paralysis of the limbs and disturbance of consciousness and was more likely to develop DVT. To date, no animal or clinical trial has been conducted to study the effect of UA on pneumonia, gastrointestinal bleeding, dysphagia, and DVT. We demonstrated that the higher UA levels (UA > 210 μmol/L) were significantly and independently a protective factor for pneumonia, poor swallow function, gastrointestinal bleeding, and DVT among ICH patients. More clinical randomized controlled trials and animal experiments are warranted to explore the influence of UA on pneumonia, dysphagia, gastrointestinal hemorrhage, and DVT after cerebral hemorrhage.

In our study, UA levels were found to have no statistically significant relationship to the non-routine disposition and in-hospital mortality at discharge in ICH patients. Similarly, a study on 221 African hemorrhagic stroke patients showed that UA levels were not an independent predictor of the mortality and adverse outcome (mRS > 2) for ICH (17). Wu et al., in a study of 380 Chinese hemorrhagic stroke patients, demonstrated that UA levels of ICH patients were not correlated with poor outcomes (mRS > 2 or death) (12). The study population in the two studies were both small, which may have affected the results. The sample size in our study was larger, but we had no discharge function outcome such as mRS score and follow-up information. More researches are still needed to explore the correlation between UA levels and function outcome of ICH.

CSCA is a nationwide, retrospective, multiple-center study in China. The inclusion of patients from different hospitals across China limited bias inherent in studies. However, there were some limitations in our study. First, the data were collected via the web-based patient data collection and management tool. The aim was to promote stroke center development and organize the delivery of stroke care. We had no access to data of the dynamic change in UA levels, and it was a pity that the use of uric acid-lowering drugs in the hospital were not collected. Second, there was no data of imaging information. Third, the study had no functional outcome at discharge such as the mRS score. Finally, the CSCA database collected only in-patient data without the follow-up information. Long-term mortality rate and function outcome could not be evaluated.

In conclusion, we found no significant evidence indicating that increased UA levels were predictive of the discharge disposition and in-hospital mortality after ICH. However, UA was a protective factor for stroke severity and in-hospital complications such as pneumonia, poor swallow function, gastrointestinal bleeding, and DVT. Further clinical randomized controlled trials and animal experiments are warranted to explore the influence of UA on stroke severity, pneumonia, dysphagia, gastrointestinal hemorrhage, and DVT after cerebral hemorrhage.

## Data Availability Statement

The datasets generated for this study are available on request to the corresponding author.

## Ethics Statement

Participating hospitals received either healthcare quality assessment and research approval to collect data in the CSCA project without requiring individual patient informed consent under the common rule or a waiver of authorization and exemption from subsequent review by their Institutional Review Board.

## Author Contributions

XL and ZC interpreted the data and drafted the manuscript. XZ and YW conceived and designed the research. HG and KY acquired and analyzed the data. RJ and ZL made critical revision of the manuscript. All authors revised and agreed to be accountable for the content of the work.

## Conflict of Interest

The authors declare that the research was conducted in the absence of any commercial or financial relationships that could be construed as a potential conflict of interest.

## Abbreviations

UA: uric acid
ICH: intracerebral hemorrhage
IS: ischemic stroke
TIA: transient ischemic attack
SAH: subarachnoid hemorrhage
BMI: body mass index
SBP: systolic blood pressure
DBP: diastolic blood pressure
LDL-C: lipoprotein cholesterol
GHb: glycated hemoglobin
FBG: fasting blood glucose
Hcy: homocysteine
Cr: creatinine
BUN: blood urea nitrogen
PLT: platelets
NIHSS: National Institutes of Health Stroke Scale
DVT: deep vein thrombosis.
